# Is traditional back translation enough? Comparison of translation methodology for an ASD screening tool

**DOI:** 10.1002/aur.2783

**Published:** 2022-08-01

**Authors:** Michaela DuBay, John Sideris, Erica Rouch

**Affiliations:** ^1^ School of Education and Human Development University of Virginia Charlottesville Virginia USA; ^2^ Chan Division of Occupational Science and Occupational Therapy University of Southern California Los Angeles California USA

**Keywords:** autism spectrum disorder, culture, hispanic or latino, language, parents, psychometrics, surveys and questionnaires

## Abstract

Early identification of autism spectrum disorders (ASD) in non‐English speaking children often relies on translation of English ASD screening measures. Most measures employ a forward‐back translation approach, despite data suggesting that this method may result in poor psychometric properties. Some studies in non‐ASD fields have suggested that a rigorous method of translation with cultural adaptation may yield better psychometric properties, but no studies in the ASD field have compared the two approaches directly. This investigation compares these two translation methodologies to determine if they produce psychometrically similar or dissimilar measures. Three hundred and eighty US‐based Spanish‐speaking caregivers of children 8–16 months old were randomly assigned to complete either a forward‐back Spanish translation or a rigorous translation with cultural adaptation of a parent‐report ASD screening tool. Measurement invariance analyses determined that the two translations were psychometrically dissimilar. Additional qualitative explanatory methods using cognitive interviews examined textual differences and participant preferences between non‐invariant items.

**Lay Summary:**

Autism screening questionnaires created in English need to be translated into other languages so non‐English speaking parents can fill them out accurately. Traditionally, researchers have not considered cultural differences when they translate these questionnaires. When we compared a direct translation to a translation with cultural adaptations, the two questionnaires were statistically different. Parents interpreted and responded to the same questions differently, depending on which version they filled out.

## INTRODUCTION

Autism spectrum disorder (ASD) is thought to affect approximately 1% of the global population (Elsabbagh et al., 2012), however health disparities experienced by many of the world's populations cause vast differences in diagnosis rates, age of diagnosis, and access to ASD‐specific services (Chamak et al., 2011; Mandell et al., 2009; Morales‐Hidalgo et al., 2018; Valicenti‐Mcdermott et al., 2012). While the average age of diagnosis is approximately 4 years in the United States (Baio et al., 2018), non‐English speaking countries often report much older average ages of diagnosis, many months or even years beyond the early intervention period (Chamak et al., 2011; Morales‐Hidalgo et al., 2018; Shrestha et al., 2019).

One common method of early identification of ASD involves using parent‐report screening instruments to investigate the presence of early behavioral ASD symptoms (Thabtah & Peebles, 2019). This method relies on parents or other caregivers to answer a series of questions about their child's behaviors. Parent‐report tools are intended to be completed independently, without support from professionals to clarify the meaning of items. Thus, the validity and reliability of parent‐report tools depends at least in part on parents' ability to interpret and respond to items accurately (DuBay & Watson, 2019). However, parents' interpretation and response to items is influenced by a variety of outside factors, including education levels, literacy levels, knowledge and perception of neurodiversity, and culture. Culture provides a lens through which parents conceptualize and interpret child behaviors (Bernier et al., 2010). A behavior that one parent perceives as typical or expected may be perceived as inappropriate or strange in another culture. This could lead respondents from diverse cultures to respond to the same instrument in distinct ways, leading to differing psychometric properties of the same instrument across cultures. Specifically, the validity or reliability of an instrument is likely to be influenced by cultural interpretations of both child behaviors and instrument wording.

Because most parent‐report screening instruments were developed in English, teams often translate them into other languages for use globally. Traditionally, translation teams have used a forward‐back (FB) methodology in which one translator translates the instrument into the new language, and a second translator then translates this new text back into the original source language. Finally, the two same language versions are compared for equivalence (original source text and back‐translated text). Some items may be re‐translated and back‐translated again as needed. Following this same language comparison step, the instrument is deemed ready for use (Soto et al., 2015). There are several studies in the field of ASD and early childhood development that suggest that this FB methodology does not effectively maintain equivalent psychometric properties across translations (Ben‐Sasson & Carter, 2012; Brennan et al., 2016; Chee & de Vries, 2021; Guiberson & Rodriguez, 2010; Guo et al., 2019; Juneja et al., 2012; Kara et al., 2014; Mohamed et al., 2016; Seung et al., 2015).

An alternative set of more rigorous translation methods that incorporate cultural adaptation procedures is becoming more common outside of the fields of ASD and early childhood development (Acquadro et al., 2008; Beaton et al., 2000; Hagell et al., 2010; Squires et al., 2013). These translation with cultural adaptation (TCA) methods consider multiple dimensions of equivalence that can exist between an instrument and its translation, such as linguistic, construct, and technical equivalence, which may more effectively maintain psychometric equivalence in the translated version (Beaton et al., 2000). This more rigorous approach involves a forward translation by a team of translators from a range of backgrounds who are trained in dimensions of equivalence, followed by an examination of the quality of this forward translation. A translation and adaptation team reviews the translations and quality checking data, then decides on a pre‐final version of the instrument. Target population members then pre‐test the pre‐final version using qualitative methods. After any needed revisions, the final version is administered to a large community sample to determine population‐specific norms and relevant psychometric properties (Beaton et al., 2000; DuBay & Watson, 2019). These procedures also generally reflect the recommendations of the International Test Commission for translating and adapting tests (ITC, 2017). Although examples in the field of ASD are still limited, a small number of research teams have begun to use this approach for screening tools or other research instruments (Cuesta‐Gomez et al., 2016; DuBay, Watson, Baranek, et al., 2021; Magán‐Maganto et al., 2020; McClure et al., 2018). There are some promising findings from studies of these translations, where screen positive rates, item‐endorsement rates, and diagnostic validity appear to closely reflect the properties of the original instrument (Cuesta‐Gomez et al., 2016; Magán‐Maganto et al., 2020; McClure et al., 2018).

When translating screening instruments for clinical or empirical use, teams must choose the most appropriate translation approach. The FB approach uses fewer resources but may fail to produce psychometrically equivalent measures. The more rigorous TCA approach may result in improved psychometric properties, but is resource intensive, including increased needs for funds, personnel, and duration of procedures. There are no direct comparisons of the two translation approaches in the ASD or child development fields to directly demonstrate that one approach yields better psychometric properties than the other. Studies from other fields are inconsistent, though comparisons of missingness and respondent preferences typically favor more rigorous TCA approaches (Da Mota Falcão et al., 2003; Hagell et al., 2010; Perneger et al., 1999). Currently, the potential impact of specific translation methodology on the psychometric properties of developmental screening tools is not yet known.

This study aims to directly examine the impact of translation methodology on the psychometric properties of an ASD screening tool. We administered two same‐language translations of the same instrument: one translated using the traditional FB approach, and the other translated using a TCA approach. We compared the two versions psychometrically, incorporating explanatory qualitative analyses, to determine if the two translation methods result in psychometrically or qualitatively distinct measures.

## METHODS

An explanatory mixed methods study with randomized controlled survey design was used to examine the impact of translation methodology on psychometric properties and respondent interpretation of an ASD screening tool. A total of 380 US‐based Latin American Spanish‐speaking caregivers of children 8–16 months of age completed one of the two Spanish versions of the *First Years Inventory v 3.1* (Baranek et al., 2013) in Spanish. Approximately half were randomly assigned to complete the FB version, while the other half completed the TCA version. Respondents were blind to study aims and their condition.

The *First Years Inventory v. 3.1* (FYIv3.1; Baranek et al., 2013) is a parent‐report ASD screening tool originally developed in English in the US. The FYIv3.1 probes the frequency of specific child behaviors using a 5‐point response scale. Preliminary data suggest the FYIv3.1 has upper bounds of a sensitivity of.4 when specificity is set to .98 for ASD in a clinical sample of 8‐ to 16‐month‐olds (J. Sideris, personal communication, Feb. 18, 2020). All participants also completed a *demographic questionnaire* to gather data on the age and sex of the child and respondent characteristics (country of origin, years living in the US, proficiency in English and Spanish, SES).

### Procedures

The TCA Spanish FYIv3.1 was developed using multiple translators from a range of backgrounds, preliminary pre‐testing and back translation for the quality assurance round, and additional pre‐testing followed be consensus meetings. See DuBay, Watson, Baranek, et al. (2021) for a complete description of this process. To create the FB Spanish translation, one professional translator translated the tool into Spanish. Another subsequently translated the Spanish version back to English. An expert in ASD then compared the English versions and items with notable differences in meaning were re‐translated by the professional translator. Once translations were complete, Spanish‐speaking caregivers were recruited primarily through letters to addresses on birth records and social media ad campaigns. For birth records mailings, records for all live births were obtained from one state Department of Health for a seven‐month period in which the children were within the target age range for the instrument (8–16 months). In order to maximize recruitment of Spanish‐speaking families, mailings were sent to all records containing “Latino or Hispanic” within the ethnicity field and a Latin American country with Spanish as a primary language in at least one parent country of origin field (n = 24,701). Mailings contained a paper copy of the FYIv3.1 version to which the participant was randomized, a return envelope, a link to the questionnaires online, and an information sheet describing the study and how to participate. Social media ads targeted Spanish‐speaking parents of children in the target age range and provided a link to the survey online, which randomized to translation condition within the platform. Approximately half of participants were recruited from each method (48.7% from mailings and 51.3% from social media).

After quantitative data analysis was complete, 14 of these respondents participated in cognitive interviews via video call. Participants were strategically chosen to stratify demographic and instrument‐related factors including child gender and age, parent gender and English proficiency, and translation condition. Twenty items showing the most metric non‐invariance in loadings between translations were included as stimuli during the interviews. For each item, interview participants were shown both translations, beginning with the translation to which they were originally randomized. Participants were asked to read the item aloud, rephrase it, and explain their response. If any misinterpretations occurred, these were then clarified by the interviewer. Participants were then shown the alternative translation, asked to identify differences between the versions and the semantic impact of those differences, and asked to choose which version they found most easy to understand.

### Data analysis

Quantitative. First, data quality was examined between groups, including completion rates, missingness and variability. For completion rates, we compared groups by response rate and rate of completing the survey after initiating it. Missingness refers to how often participants leave items blank. Items left blank are a possible indication of confusion among respondents and can impede accurate score calculation (DuBay & Watson, 2019). Variability across response options both for individual items and across the entire scale were also examined. Second, measurement invariance was examined between groups. If a scale is shown to have measurement invariance when administered across different groups, this indicates that the results of the scale are solely dependent on the participants' actual level of latent variable rather than their group status (Van De Schoot et al., 2015). In this case, if measurement invariance is evident between the two conditions, this would indicate that the two translation methods produced psychometrically equivalent scales. If measurement invariance is *not* found to be maintained between the two versions, this would suggest that the dissimilar translation methods led to a divergent pattern of interpretations and/or responses to particular items or the scale as a whole. A multi‐group confirmatory factor analysis (CFA) was used to examine measurement invariance in MPlus (Muthén & Muthén, 2011). To begin, we examined the entire dataset for overall fit with a seven‐factor model previously identified for the FYIv3.1 (Baranek et al., 2022). Then, the two groups were compared using configural, metric, and scalar models. To identify potentially non‐invariant items, we examined individual items for differences in loadings and thresholds between groups as a factor of the standard error. Additional metric and scalar models were run with freed factor loadings and thresholds for items with the greatest differences.

Qualitative. MD and ER examined data from all participants across items. For each item, discrepancies in participant interpretation and intended meaning were compiled across participants. Patterns of errors were then compared across translation conditions. We examined specific differences and larger patterns in participants' textual comparisons between translations. Finally, participant preference for translation condition was compiled across all items.

## RESULTS

### Participants

A total of 380 caregivers completed the FYIv3.1, with 192 in the TCA condition and 188 in the FB condition. Respondents mainly consisted of mothers (93.6%), with several fathers (5.4%) and a small number of other caregivers (e.g. grandparents, aunts). Respondents reported a wide range of countries of origin, including almost all Latin American countries where Spanish is considered an official language (See Table A1, Appendix). Of those reporting a country of origin outside the US, respondents had lived in the US for an average of 10.2 years (range 1–36 years). While all respondents reported native Spanish proficiency, they reported a range of English proficiencies, household incomes, and education levels (See Table A2, Appendix). Respondents ages ranged from 18 to 64 years, with an average age of 31.0 years. Children ranged in age from 8 to 16 months, with an average age of 11.3 months. Child genders were represented fairly evenly (48.3% girls). The percentage of children who scored above the likelihood threshold in both social communication and sensory reactivity was 7.9%. There were no differences between groups in child gender, age, or likelihood status.

### Data quality

*Completion*. Response rate was compared between groups on mailed surveys. The response rate was similar across families who were mailed the TCA version (1.40%) and the FB version (1.37%). (See Brown et al. (2014) for a discussion of recruitment challenges in this population.) Completion rates were examined among participants who completed the survey online only, as all surveys returned by mail were returned completed. Completion rate was defined as the percentage of participants who answered the last FYIv3.1 question out of the total number of participants who answered at least the first FYIv3.1 question. Completion rate was 86.7% for those randomized to the TCA group and 96.7% for those randomized to the FB group. This difference was statistically significant (*χ* ^2^ = 5.97, *p <* 0.05), indicating that those who responded to the TCA version were more likely to discontinue the survey prior to completion.

*Missingness*. The total number of items either left blank or where more than one response option was selected was examined, excluding those who discontinued the survey prior to completing 75% of the items. Missingness was not significantly different between groups (*t =* 1.2, *p =* 0.22).

*Variability*. A *χ* ^2^ analysis indicated that respondents in the two translation conditions selected response options using significantly different patterns (*χ* ^2^ = 224.23, *p <* 0.0001). See Table A3 (Appendix) for proportions of response option selection between groups. A residual analysis of the *χ* ^2^ results revealed that the TCA group chose the second response option with far greater frequency than the FB group (TCA = 6.82, FB = −6.86), while the FB group chose the fifth response option with far greater frequency than the TCA group (TCA = −6.40, FB = 6.44). All residuals were either >2 or < −2, indicating a significant deviation from expectation between groups for all response options. Comparing the frequency of choosing each response category, the TCA group ranged from 16.3% to 24.8% across the 5 categories, while the FB group ranged from 11.8% to 30.7%. This indicated a more evenly distributed selection of response options among the TCA group as compared to the FB group. Post hoc analyses comparing the frequency of choosing extreme response options (first and fifth options) as well as the frequency of choosing intermediate response options (second and fourth options) between groups were both significant (*χ* ^2^ = 184.48, *p <* 0.0001 and *χ* ^2^ = 135.89, *p <* 0.0001, respectively).

### Measurement invariance

The seven‐factor CFA showed reasonable fit in the full sample (see Table A4 (Appendix) for all model fit indices, including *χ* ^2^, *χ* ^2^ degrees of freedom, RMSEA or root mean square of approximation, and CFI or comparative fit index). Next, a configural model was run comparing model fit for each group with no constraints, which revealed good fit. Factor loadings were then fixed between the two groups to run a metric model. This model showed a reduction in fit and was significantly different than the configural model (*χ* ^2^ = 115.62, *p <* 0.0001; see Table A5 (Appendix) for all model fit comparison analyses). Thus, non‐invariance was identified at this first level of invariance testing, such that item factor loadings were not found to be equivalent between groups.

To identify which items may be most non‐invariant, we examined item differences between the two groups in the configural model. For each item, we computed the absolute value of differences and the average standard of the factor loadings between the two groups to explore what magnitude of difference in factor loadings contributed to non‐invariance. Twenty items had differences more than 0.1 beyond the average standard error (see Table A6, Appendix). Next, we tested a partial metric model with freed factor loadings for these 20 items to determine if fit improved. This metric model did not differ significantly from the configural model (*χ* ^2^ = 29.42, *p =* 0.93), suggesting that factor loadings on the other 49 items can be constrained to equality between groups. This finding also indicated that the 0.1 criterion successfully identified a sufficient number of items that contributed to non‐invariance. The percentage of these non‐invariant items varied widely across the factors: 0% of items in the Milestones/Motor Coordination (MCM) factor, 13% of Self‐regulation in Daily Routines (SDR), 17% of Communication, Imitation, and Play (CIP), 29% of Social‐Affective Attention & Engagement (SAAE), 43% of Hyper‐Responsivity, 63% of Hypo‐Responsivity, 71% of Sensory Interests, Repetitions, and Seeking behaviors (SIRS).

Additional constraints were then added to the model by fixing item intercepts, or thresholds, between the two groups. This scalar model showed decreasing fit and differed significantly from the partial metric model (*χ* ^2^ = 200.73, *p <* 0.0001). Thus, item thresholds were not found to be equivalent between groups, additionally contributing to non‐invariance.

To examine which items may have been contributing to scalar non‐invariance, a similar procedure was conducted for the item thresholds. Using the metric model, the absolute value of the difference in item thresholds between groups were compared to the average standard error. There were 17 items with a difference in thresholds greater than 0.15 beyond the average standard error (see Table A7, Appendix). Six of these items overlapped with those most non‐invariant in loadings. The partial metric model was extended to allow for scalar non‐invariance in thresholds for these 17 items. The scalar model did not differ significantly from the configural model (*χ* ^2^ = 77.997, *p* = 0.88), or from the metric model (*χ* ^2^ = 48.577, *p* = 0.61), suggesting that thresholds on the other 52 items appeared to be equivalent between groups and that the.15 criterion was sufficient. As with the factor loadings, there was no evidence of invariance on the MCM factor. Across the remaining factors, 13% of Hypo‐Responsivity, 13% of SDR, 29% of SIRS, 29% of SAAE, 29% of Hyper‐Responsivity, and 39% of CIP fell above the 0.15 criterion.

Overall, for the SDR factor, 25% of items showed non‐invariance in either factor loadings or thresholds. This percentage was 43% in Hyper‐Responsivity, 44% in CIP, 50% in SAAE, 63% in Hypo‐Responsivity, and 86% in SIRS (with only one non‐invariant item). Variability of selection of response options differed significantly between the groups on 17 of these 31 items (*χ* ^2^ ranged between 9.68 and 38.89, *p*‐values ranged between <0.0001 and <0.05). Proportion of missingness between groups did not differ significantly on any of the 31 items.

### Qualitative findings

Fourteen survey respondents also completed a qualitative interview, with equal representation from each translation condition. Average age of the child at the time of the survey was 11.4 months (range 8–16 months). Gender was also evenly represented in the sample of children; however, respondents were 13 mothers and one father. Eight children had scored below the likelihood threshold in both domains, three had scored above the threshold in one domain, and three had scored above the threshold in both domains. Compared to the survey sample, the interview sample had a somewhat greater representation of participants with higher English proficiency and higher education levels, greater representation from Central America and no one from the US, and participants had been living in the US on average 7.4 years (range 2–36 years). Participants reviewed the 20 items that differed the most at the factor loadings level.

Overall, the frequency of misinterpretations was similar between the TCA and FB groups. Examples of misinterpretations included omission of a section of the question, unfamiliarity with specific terms, or subject/object confusion. When presented with both versions, participants preferred the TCA version 63% of the time. There were 7 items where participants reported a strong preference for the TCA version (at least 70% of participants preferred), 2 items where participants reported a strong preference for the FB version, and 11 items without a strong preference. For participants who experienced a misinterpretation, they also preferred the TCA version 63% of the time after fully understanding the intent of the item.

When comparing text between items, participants agreed upon preference for several specific terms, often related to actions and descriptive terms. Participants overwhelmingly chose the term used in the TCA version 67% of the time and the term used in the FB version 33% of the time. There were 5 terms that at least 1 participant was unfamiliar with, 4 of which were used in the FB version and 2 of which were used in the TCA version. Participants also noted several differences in word order within the text, preferring the TCA version 67% of the time. Generally, participants reported that they preferred items to be short, simple, and direct. This meant that some participants considered examples or further clarification within an item to be unnecessary, only adding to the length and complexity of the text. However, others noted the need for the additional details, stating that they were crucial to understand the question, despite the fact that they increased the length. Participants universally agreed that including two genders (hijo/hija) within the items was preferred to just one (hijo).

## DISCUSSION

The purpose of this explanatory mixed methods study was to compare two methods of translation of an ASD screening tool to directly examine how translation methods impact response patterns and psychometric properties of an instrument. Using CFA to examine measurement invariance, the two measures were determined to be non‐invariant, or not psychometrically equivalent. Interview data revealed qualitative differences in interpretations and response patterns between the two versions. Thus, the difference in translation methods significantly impacted the way respondents interpreted and responded to items, which impacted the psychometric properties of this ASD screening tool.

### Data quality and completeness

The literature in translation methodology recommends special attention to translation of response options to avoid patterns of response bias (Beaton et al., 2000; Stewart & Napoles‐Springer, 2000; Ware et al., 1996; Ware & Gandek, 1998) In this study, there were differences in the selection of response options across the range of responses between groups. Overall, The TCA group's choice of response options was more evenly distributed across all options as compared to the FB group. The FB group was more likely to choose extreme responses (either “always” or “never”) than the TCA group and was less likely to choose intermediate response options (second and fourth options). Considering the textual differences between the versions, these findings were expected. First, response options in the TCA version were labeled with a translation of “yes” or “no” at the beginning of each response option, while the FB version, which was a more direct translation, did not make this addition. This addition was chosen because qualitative data used in the translation of this version identified a tendency to misunderstand frequency‐based response options in the context of the yes/no questions used in the FYIv3.1 (DuBay, Watson, Baranek, et al., 2021). Given the phrasing of the questions, respondents may only have considered “always” or “never” as valid responses. Including a “yes” or “no” prior to the five response categories may have opened up the middle three responses as acceptable responses for participants. Second, the TCA version labeled each of the five response options, while the FB version maintained a lack of labels for the intermediate options (two and four), as in the original English version. This difference may have led to the FB group being less likely to choose unlabeled response options if they were confused about the meaning of these response options. This pattern was also identified in qualitative cognitive interviews conducted during the translation of the TCA version of this instrument (DuBay, Watson, Baranek, et al., 2021).

Instructions are also an area of importance but are frequently overlooked in translation of research and clinical instruments (Acquadro et al., 1996; Guillemin, 1993; Sousa & Rojjanasrirat, 2011). Due to unintended interpretations and responses from cognitive interview participants in DuBay, Watson, Baranek, et al. (2021) , the TCA translation added this statement at the end of the instructions: “While we review your responses, we will consider your child's age. How old is your child now (in months)?/*Mientras revisamos sus respuestas, vamos a considerar la edad de su niño/niña*. *¿Qué edad tiene su niño/niña ahora (en meses)?*” The child's age is already calculated based on the respondent's report of the child's birthdate and date of response. This additional question was added to encourage participants to report accurate behavior frequencies instead of adjusting reported frequencies based on their own expectations about the child's developmental age. The finding that FB respondents, who were not primed with this additional question, chose “always” more often than the TCA group may have been a result of respondents reporting higher levels of skills than were accurate because of this upward adjustment of response options based on expectations for their child's age.

Textual differences in instructions and labels of response options appeared to have resulted in response bias in this sample. In instruments related to child development, socially desirable responding and a tendency to choose extreme response categories are likely patterns (Stewart & Napoles‐Springer, 2000; Taylor et al., 2014), and ones that may have been evident here, especially in the FB version. These response bias patterns may affect the accuracy of both norm development and calculations of raw scores on an individual basis.

There were similar rates of respondents leaving items blank across groups, and similar response rates for those completing the survey on paper. However, respondents randomized to the TCA group were more likely to discontinue the survey prior to completion as compared to the FB group. Items left blank, including on uncompleted screeners, inhibits the calculation of an accurate likelihood score (DuBay & Watson, 2019; Stewart & Napoles‐Springer, 2000). The additional length of the TCA survey due to the additional question within the instructions, additional labels on response options, and extra explanations within some items to increase intelligibility may have increased the overall burden for this group and led to a lower completion rate. This finding was further supported qualitatively within the preferences for shorter text within items. The TCA version contained a total of 1,524 words, while the FB version contained 1,261 words. Completion rates, along with intelligibility of the instrument, will also influence the accuracy of norms as well as individual scores.

### Measurement invariance

When testing the 7‐factor structure with each group with no constraints, the proposed structure showed good fit. However, metric non‐invariance was identified at the next level of invariance testing such that factor loadings were not found to be equivalent between groups. This suggests that the items in a given factor do not provide the same contribution to that construct between translation versions of the instrument (Putnick & Bornstein, 2016). This indicates that the method of translation had an impact on the relationship between those items and the factor they are intended to represent. Twenty items appeared to show the greatest differences in factor loadings between the two groups. Freeing loadings for these items did improve fit for the metric model, however scalar non‐invariance was also identified at the following level, where item intercepts were not found to be equivalent between groups. This suggests that respondents with the same level of latent variable did not respond to items in the same way across translation versions of the instrument (Bialosiewicz et al., 2013). Seventeen items (6 of which overlapped with the previous 20) appeared to show the greatest differences in thresholds between the two groups. Freeing thresholds for these items similarly improved fit for the scalar model. Most of the non‐invariant items were found in factors related to restrictive and repetitive behaviors, with a significant number in factors related to social communication. Most items related to daily routines and motor skills were invariant, or psychometrically similar. Social communication behaviors are thought to vary significantly cross‐culturally (Mandy et al., 2014), however the cultural conceptualization of descriptions of restrictive and repetitive behaviors may have influenced interpretation of these items more so than those related to social communication. Culture may have less influence on conceptualization of motor skills and daily routines in this context or within the cultures represented here.

It is unknown whether differences in other items, response options, or instructions may have also contributed to non‐invariance (Acquadro et al., 1996; Guillemin, 1993; Sousa & Rojjanasrirat, 2011). A qualitative study of the TCA translation process for this instrument did in fact identify a range of unintended interpretations and response patterns from participants within almost all FYI items, response options, and instructions (DuBay, Watson, Baranek, et al., 2021).

### Qualitative findings

The most non‐invariant 20 items in factor loadings were examined via cognitive interviews among 14 participants who had responded to the survey. Results indicated a moderate preference for the TCA version, which is supported by previous literature (Hagell et al., 2010). When participants noted specific textual differences between the terminology and syntax across items, they often preferred the TCA version. However, misinterpretations happened as frequently in the TCA version as they did in the FB version, and the increased length of the TCA version may have led to lower completion rates for this version.

### Limitations

Diagnostic outcome data was not available due to the age of the children in the sample, precluding a comparison of sensitivity or specificity rates between translation versions. While the two instruments appear to differ psychometrically, it is not known with certainty whether this non‐invariance contributes to clinically significant differences in validity or reliability. For the qualitative data, our representation of those who scored above thresholds in one or two domains was higher than the rate within the larger sample. However, this included data from only 3 participants whose children scored above the threshold in both domains, limiting the variability within this sample. Interpretation patterns may differ among children who are in fact at higher likelihood of having ASD or another neurodevelopmental disorder as compared to typically developing children. Participants responded in two ways (paper/pencil and online), which may have differentially influenced response patterns; however, we were not able make comparisons based on format of administration. Finally, there may have been differences in interpretation and response patterns according to differences in Spanish dialects or Latin cultures, as many diverse groups were represented in the sample. There was not sufficient representation of various dialects or cultures to complete within‐group comparisons either quantitatively or qualitatively.

## CONCLUSIONS

The identified non‐invariance and explanatory qualitative findings here indicate that translation methods significantly impact an instrument's psychometric properties. The way that an item is worded influences how parents and caregivers interpret and therefore respond to the item. The way instructions are presented and the way respondents are expected to answer items may also systematically influence response patterns. Most differences were identified in items related to restrictive and repetitive behaviors, as well as many items related to social communication behaviors. While there were similar rates of misinterpretations between translation versions, participants reported moderate preference for the TCA version. Variability of responses was also greater within this version, through its increased length may have led to lower completion rates.

Future research should compare the diagnostic validity and reliability between instruments using distinct translation methods to determine the exact clinical implications of different approaches. Additionally, examining measurement invariance between these FYIv3.1 translations and the original English version of the FYIv3.1 could provide evidence for which method appears to result in a more psychometrically equivalent translation.

## FUNDING INFORMATION

University of Virginia Supporting Transformative Autism Research (Grant number: Pilot Award).

## ETHICS STATEMENT

All procedures were prospectively reviewed and approved by an ethics committee (the University's Institutional Review Board).

## Data Availability

The data that support the findings of this study are available from the corresponding author upon reasonable request.

## Appendix

**TABLE A1:** Reported countries of origins of respondents

Region	Country	*N*	Percentage of total
*North America*	Mexico	80	22.0%
	United States	64	17.6%
*Central America*	El Salvador	48	13.2%
	Honduras	33	9.1%
	Guatemala	28	7.7%
	Nicaragua	4	1.1%
	Costa Rica	2	0.6%
	Panama	1	0.3%
*Caribbean*	Puerto Rico	20	5.5%
	Cuba	13	3.6%
	Dominican Republic	8	2.2%
*South America*	Colombia	28	7.7%
	Venezuela	11	3.0%
	Peru	10	2.8%
	Bolivia	8	2.2%
	Chile	3	0.8%
	Argentina	1	0.3%
	Ecuador	1	0.3%
	Paraguay	1	0.3%

*Note*: Some respondents specifically reported “Puerto Rico” as their country of origin. While Puerto Rico is a territory of the United States, this origin was retained separately for the participants that self‐reported this as Puerto Rico has a unique dialect compared to dialects spoken in other areas of the United States. Sixteen participants did not indicate their country of origin. There were no differences between groups in country of origin.

**TABLE A2:** Socio‐economic variables of respondents, according to translation condition

	FB condition	TCA condition	Total
*Level of English proficiency* (*i*.*e*., “*Do you speak English?*”)			
“Very well”	39.5%	37.0%	38.2%
“Well”	17.8%	16.8%	17.3%
“Somewhat”	10.3%	9.8%	10.0%
“A little”	14.0%	14.7%	14.4%
“Not much”	18.4%	21.7%	20.1%
*Annual household income* (*USD*)			
$19,999 or less	22.4%	25.2%	23.8%
$20,000–39,999	28.2%	27.0%	27.6%
$40,000–59,999	11.6%	15.1%	13.3%
$60,000–79,999	14.8%	10.1%	12.4%
$80,000–99,999	9.6%	6.9%	8.3%
$100,000–149,999	5.1%	9.4%	7.3%
$150,000 or more	8.3%	6.3%	7.3%
*Highest education level achieved*			
8th grade or less	9.9%	8.8%	9.4%
Some high school	5.0%	7.7%	6.3%
High school diploma or equivalent	12.1%	13.3%	12.7%
Some college	22.1%	25.4%	23.8%
Bachelor's degree or equivalent	26.0%	21.6%	23.8%
Master's degree or more	24.9%	23.2%	24.0%

*Note*: All group differences in demographic factors were non‐significant.

**TABLE A3:** Frequency of overall selection of each response option by respondents, according to translation condition

	TCA condition	FB condition
1 “Never” in original version	18.8%	21.3%
	“No, nunca”	“Nunca”
2 Not labeled in original version	16.3%	11.8%
	“No, casi nunca”	Not labeled
3 “Sometimes” in original version	23.5%	21.7%
	“Sí, a veces”	“A veces”
4 Not labeled in original version	16.6%	14.5%
	“Sí, casi siempre”	Not labeled
5 “Always” in original version	24.8%	30.7%
	“Sí, siempre”	“Siempre”

**TABLE A4:** Model fit for configural, metric, scalar, and partial models

	*Χ* ^2^	*Χ* ^2^ DF	RMSEA	CFI
Configural model	7657.47	4512	0.061	0.624
Full metric model	7773.08	4574	0.061	0.618
Full scalar model	7976.49	4643	0.061	0.601
Partial metric model^a^	7686.89	4554	0.061	0.624
Partial scalar model^b^	7735.46	4606	0.060	0.626

^a^ Factor loadings freed for 20 most non‐invariant items in loadings.

^b^ Factor loadings freed for 20 most non‐invariant items in loadings and intercepts freed for 17 most non‐invariant items in thresholds.

**TABLE A5:** Comparisons in fit between models

	*Χ* ^2^	*Χ* ^2^ DF	*p*
Configural versus Full metric	115.62	62	<0.0001
Full metric versus Full scalar	203.41	69	<0.0001
Configural versus Full scalar	319.02	131	<0.0001
Configural versus Partial metric^a^	29.42	42	0.9286
Partial metric^a^ versus Full scalar	200.73	69	<0.0001
Configural versus Partial scalar^b^	78.00	94	0.8833

^a^ Factor loadings freed for 20 most non‐invariant items in loadings.

^b^ Factor loadings freed for 20 most non‐invariant items in loadings and intercepts freed for 17 most non‐invariant items in thresholds.

**TABLE A6:** Comparison of item factor loadings between groups, as a factor of the average standard error

Factor	Item number	DFL	ASE	DFL–ASE
Communication, imitation & play	2	0.025	0.100	−0.075
	13	0.021	0.080	−0.059
	14	0.085	0.073	0.012
	18	0.022	0.071	−0.049
	21	0.164	0.086	0.079
	23	0.021	0.098	−0.077
	25	0.055	0.074	−0.019
	29^a^	0.230	0.087	0.144
	33	0.062	0.089	−0.027
	39	0.047	0.096	−0.049
	41	0.157	0.092	0.066
	43	0.123	0.079	0.045
	44	0.057	0.084	−0.027
	45^a^	0.247	0.080	0.168
	53^a^	0.235	0.071	0.165
	57	0.022	0.097	−0.075
	60	0.114	0.080	0.034
	65	0.071	0.077	−0.006
Social‐affective attention & engagement	1^a^	0.180	0.058	0.123
	3	0.055	0.073	−0.018
	8^a^	0.158	0.048	0.111
	9^a^	0.172	0.061	0.112
	22	0.155	0.067	0.089
	27	0.079	0.095	−0.016
	34	0.101	0.097	0.004
	35	0.132	0.056	0.076
	36	0.159	0.077	0.082
	49	0.013	0.065	−0.052
	58	0.120	0.057	0.064
	61^a^	0.259	0.069	0.190
	64	0.049	0.062	−0.013
	66	0.061	0.078	−0.017
Hyper‐responsivity	10	0.094	0.101	−0.007
	17	0.106	0.0995	0.006
	19^a^	0.26	0.089	0.171
	32	0.093	0.0825	0.011
	46	0.112	0.0915	0.021
	56^a^	0.26	0.084	0.176
	62^a^	0.208	0.0985	0.110
Hypo‐responsivity	7	0.019	0.091	−0.072
	15^a^	0.384	0.088	0.296
	26	0.054	0.069	−0.015
	30^a^	0.373	0.074	0.300
	31	0.146	0.045	0.102
	40^a^	0.344	0.076	0.268
	47^a^	0.229	0.091	0.138
	51	0.096	0.082	0.015
Self‐regulation in daily routines	6	0.102	0.090	0.013
	12	0.098	0.074	0.024
	16	0.133	0.074	0.059
	28	0.163	0.098	0.065
	38	0.047	0.083	−0.036
	42^a^	0.255	0.110	0.145
	67	0.041	0.080	−0.039
Sensory interests, repetitions, and seeking behaviors	4	0.047	0.106	−0.059
	11^a^	0.216	0.091	0.125
	20	0.128	0.099	0.029
	37^a^	0.554	0.102	0.453
	52	0.023	0.081	−0.058
	59^a^	0.215	0.085	0.130
	63^a^	0.288	0.094	0.195
Milestones/Motor coordination	5	0.053	0.070	−0.017
	24	0.050	0.066	−0.016
	48	0.023	0.083	−0.060
	50	0.029	0.058	−0.029
	54	0.104	0.092	0.012
	55	0.026	0.086	−0.060
	69	0.059	0.107	−0.048

Abbreviations: ASE: average standard error of factor loadings between groups; DFL: difference in factor loadings between groups.

^a^ Items with DFL‐ASE >0.1.

**TABLE A7:** Comparison of item thresholds between groups, as a factor of the average standard error

Factor	Item number	DTh	ASE	DTh–ASE
Communication, imitation & play	2^a^	0.284	0.106	0.178
	13	0.155	0.088	0.067
	14	0.022	0.077	−0.055
	18^a^	0.246	0.076	0.170
	21	0.149	0.088	0.062
	23	0.140	0.106	0.035
	25^a^	0.350	0.081	0.270
	29	0.037	0.098	−0.061
	33	0.132	0.098	0.035
	39^a^	0.288	0.108	0.181
	41	0.230	0.100	0.131
	43	0.077	0.080	−0.003
	44	0.109	0.091	0.019
	45^a^	0.437	0.087	0.350
	53^a^	0.422	0.077	0.346
	57^a^	0.399	0.109	0.291
	60	0.183	0.087	0.097
	65	0.177	0.083	0.095
Social‐affective attention & engagement	1	0.115	0.058	0.057
	3^a^	0.303	0.071	0.232
	8^a^	0.245	0.047	0.199
	9	0.044	0.060	−0.016
	22^a^	0.280	0.066	0.214
	27	0.100	0.087	0.013
	34	0.109	0.092	0.017
	35	0.009	0.057	−0.048
	36	0.149	0.073	0.076
	49	0.029	0.064	−0.035
	58	0.007	0.057	−0.050
	61	0.201	0.072	0.130
	64	0.098	0.062	0.036
	66^a^	0.279	0.077	0.203
Hyper‐responsivity	10	0.060	0.087	−0.027
	17	0.145	0.089	0.057
	19	0.031	0.078	−0.047
	32	0.083	0.076	0.008
	46	0.033	0.080	−0.047
	56^a^	0.300	0.076	0.225
	62^a^	0.549	0.087	0.463
Hypo‐responsivity	7	0.079	0.084	−0.005
	15	0.121	0.084	0.037
	26	0.006	0.064	−0.058
	30^a^	0.247	0.067	0.180
	31	0.002	0.043	−0.041
	40	0.186	0.064	0.122
	47	0.113	0.082	0.031
	51	0.112	0.076	0.037
Self‐regulation in daily routines	6	0.076	0.081	−0.005
	12^a^	0.298	0.065	0.234
	16	0.175	0.066	0.109
	28	0.127	0.087	0.040
	38	0.07	0.077	−0.007
	42	0.046	0.095	−0.049
	67	0.052	0.074	−0.022
Sensory interests, repetitions, and seeking behaviors	4	0.069	0.093	−0.024
	11	0.076	0.082	−0.006
	20^a^	0.297	0.094	0.204
	37	0.168	0.093	0.076
	52^a^	0.507	0.073	0.434
	59	0.136	0.074	0.063
	63	0.198	0.089	0.110
Milestones/Motor coordination	5	0.116	0.067	0.049
	24	0.017	0.060	−0.043
	48	0.210	0.079	0.131
	50	0.037	0.055	−0.018
	54	0.078	0.089	−0.011
	55	0.096	0.084	0.012
	69	0.197	0.094	0.103

Abbreviations: ASE: average standard error of thresholds between groups; DTh: difference in thresholds between groups.

^a^ Items with DTh‐ASE >0.15.
